# Aislamiento y confinamiento: La otra pandemia en el ámbito social*

**DOI:** 10.15649/cuidarte.2124

**Published:** 2023-05-30

**Authors:** Andrea Hernández Quirama, Hector Mauricio Rojas Betancur, Johana Linares García

**Affiliations:** 1 Universidad Industrial de Santander, Bucaramanga, Colombia. Email: ahernanq@uis.edu.co Universidad Industrial de Santander Universidad Industrial de Santander Bucaramanga Colombia ahernanq@uis.edu.co; 2 Universidad Industrial de Santander, Bucaramanga, Colombia. Email: hmrojasb@uis.edu.co Universidad Industrial de Santander Universidad Industrial de Santander Bucaramanga Colombia hmrojasb@uis.edu.co; 3 Universidad Industrial de Santander, Bucaramanga, Colombia. Email: johana.linares.garcia@gmail.com Universidad Industrial de Santander Universidad Industrial de Santander Bucaramanga Colombia johana.linares.garcia@gmail.com

**Keywords:** Interacción Social, Pandemia, Familia, Confinamiento, Aislamiento, Social Interaction, Pandemic, Family, Confinement, Isolation, Interação Social, Pandemia, Família, Confinamento, Isolamento

## Abstract

**Introducción:**

El aislamiento y el confinamiento son medidas de alto impacto social que, a nivel mundial y en mayor o menor grado de intensidad, han provocado cambio, temporales o permanentes, respecto a la forma en que se realizan las interacciones sociales.

**Objetivo:**

realizar un estudio en el ámbito de lo doméstico y de las modificaciones externas que obligaron a un encierro drástico en la población colombiana en los primeros meses de la pandemia por la COVID-19.

**Materiales y métodos:**

Se realizó un estudio con enfoque cualitativo a partir de la aplicación de entrevistas en profundidad a 45 participantes residentes en la ciudad de Bucaramanga, Colombia, logrando la reconstrucción de rutas de vida para evaluar el momento más drástico del encierro de la población.

**Resultados:**

Desde los hallazgos, se resalta la capacidad de asimilación de las fuertes medidas de confinamiento, la colectivización de las justificaciones más legitimadas desde el discurso médico y la construcción de medidas de autocuidado constituidas en el ámbito de lo doméstico como lugar y espacio relacional clave para enfrentar la incertidumbre social frente a la ausencia de respuestas efectivas para el control del contagio y de la enfermedad.

**Conclusión:**

El drástico encierro de la población colombiana a partir de las medidas transitorias, conlleva una alta incertidumbre de los grupos familiares, pero también una respuesta generalmente positiva.

## Abstract (en)

**Introduction:**

Isolation and confinement are measures of high social impact that -worldwide and to a greater or lesser degree of intensity- have brought about temporary or permanent changes in how social interactions are conducted.

**Objective:**

To conduct a study on the domestic sphere and the external modifications that forced a drastic lockdown in the Colombian population during the first months of the COVID-19 pandemic.

**Materials and Methods:**

A qualitative study was carried out by conducting in-depth interviews with 45 participants living in the city of Bucaramanga, Colombia, reconstructing life pathways to evaluate the most drastic moment in the population’s lockdown.

**Results:**

From the findings, the ability to assimilate strong lockdown measures, the collectivization of the medical discourse’s most legitimate justifications, and the construction of self-care measures in the domestic sphere -a key place and relational space to face social uncertainty in the absence of effective responses to control contagion and disease- are highlighted.

**Conclusion:**

The drastic lockdown of the Colombian population due to the transitory measures entailed a high level of uncertainty for family groups but also a generally positive response.

## Resumo

**Introdução:**

Isolamento e confinamento são medidas de alto impacto social que, em todo o mundo e em maior ou menor grau de intensidade, têm causado mudanças temporárias ou permanentes na forma como as interações sociais são realizadas.

**Objetivo:**

realizar um estudo no âmbito doméstico e as modificações externas que forçaram um confinamento drástico na população colombiana nos primeiros meses da pandemia de COVID-19.

**Materiais e Métodos:**

Realizou-se um estudo com abordagem qualitativa a partir da aplicação de entrevistas em profundidade a 45 participantes residentes na cidade de Bucaramanga, Colômbia, realizando a reconstrução de rotas de vida para avaliar o momento mais drástico do confinamento de a população.

**Resultados:**

A partir dos achados, destaca-se a capacidade de assimilação das medidas de confinamento forte, a coletivização das justificativas mais legítimas do discurso médico e a construção de medidas de autocuidado constituídas na esfera doméstica como lugar e espaço relacional fundamental. enfrentam a incerteza social na ausência de respostas eficazes para controlar o contágio e a doença.

**Conclusão:**

O confinamento drástico da população colombiana como resultado das medidas transitórias acarreta um alto grau de incerteza para os grupos familiares, mas também uma resposta geralmente positiva.

## Introducción

El aislamiento, el confinamiento y, además, el distanciamiento, son tres medidas generalizadas y consideradas necesarias desde la lógica de contención de la pandemia por la COVID-19, un virus que ha afectado a todas las sociedades y los individuos. La ausencia de una respuesta efectiva desde el punto de vista médico y epidemiológico en los primeros meses de la pandemia^1^^,^^2^, ha intensificado la prolongación de medidas de contención, al menos en lo trascurrido en año 2020 y el inicio del 2021, aunque se avanza en el proceso general de inoculación con diferentes vacunas y tratamientos farmacológicos.

Cerrar en alto grado las sociedades, las economías, el tránsito y la interacción social^3^, constituye la otra pandemia con consecuencias diferenciadas en grado e intensidad, pero a una escala sin precedentes a nivel mundial y extendida en prácticamente todos los niveles, países y estratos sociales.

Las medidas de cierre de las interacciones sociales más próximas al individuo, como su posibilidad de desarrollar las actividades cotidianas en referencia con las relaciones económicas, educativas y sociales, implican cambios en el significado mismo de la vida en sociedad, el hecho coyuntural del encierro en los hogares y en pequeños núcleos familiares, reduce el ámbito de posibilidades de reproducción de la vida en sociedad^4^, pero está justificado en el pleno cierre de los grandes sistemas sociales, las instituciones y las funciones que cotidianamente configuran el ritmo de la vida cotidiana.

Las drásticas medidas por la pandemia, es la suspensión momentánea, pero abrupta, de la participación social, aunque, simbólicamente, esa participación se traslada al uso de tecnologías de la comunicación^5^, deja una sensación de ruptura, de crisis y de cambio que se debe asumir en el lugar al que trasladamos una condición de aparente seguridad cuando no existió otro camino que reducir casi totalmente la circulación de nuestros intereses, medidas que también han sido criticadas desde la propia academia^6^.

El hogar tuvo que convertirse no solo en el microescenario de las interacciones, también tuvo que asumirse como el lugar seguro, el único, como la pantalla protectora que afirma la minimización del riesgo de contagio y de enfermedad^7^. Pero el hogar no es una categoría homogénea y tampoco significa lo mismo para toda la población, no sólo por las diferencias materiales, las posibilidades de acceso a bienes de consumo, sino, además, por las configuraciones tan variadas y complejas alrededor de las relaciones entre los integrantes de esos hogares.

Por una parte, el encierro obligado y obligatorio de gran parte de la población en esos micro escenarios, tiene consecuencias diversas en las personas y en los sistemas sociales^8^, tanto a nivel de las afectaciones en salud como en las prácticas de acomodamiento y supervivencia, así como en cambios temporales y permanentes en la manera en que entendemos y asumimos la normalidad social. Por otra parte, las medidas de salud pública que afectan de manera importante los flujos de interacción social, se han presentado y legitimado como transitorias, bajo la promesa de un retorno a cierto estado de normalidad^9^.

Este marco de legitimaciones lleva implícito el poder del discurso médico: se toman medidas políticas respaldadas por la autoridad del conocimiento sanitario, recurriendo al discurso de la historia de las pandemias y a la figura de un proceso inédito para la sociedad, explotando la capacidad de las personas de asimilar la necesidad, pero también la obediencia, de las diferentes medidas públicas para contrarrestar escenarios, reales o imaginarios, nefastos para la seguridad individual y colectiva^2^.

Frente a la inminencia del contagio, la enfermedad y la muerte, aunque sea baja la probabilidad matemática, se colectiviza el miedo y la sensación de autorresponsabilidad en la mayor parte de la población, de hecho, la desobediencia de las normas generales y de autocuidado, ha constituido una moneda de cambio para la sanción al comportamiento no adecuado en medio de la pandemia, como una manera de hacer una pedagogía del riesgo que garantice el mantenimiento de las medidas y el retorno continuo a procesos de cierres temporales y territoriales, dados los datos dinámicos de velocidad de los contagios, las variantes del virus y la incapacidad, aún, de contrarrestar médicamente las consecuencias del virus a nivel global.

Una consecuencia interesante de la pandemia del COVID-19, es la pandemización de la sensación de estar inmersos en un proceso global, que nos afecta a todos en el planeta y que, de muchas maneras, todos somos responsables del origen y de las soluciones. Responsabilidad por el mal manejo ambiental, por el ritmo acelerado de las formas de vida y de consumo, de movilidad y de conectividad, sumado al individualismo y el egoísmo en nuestras acciones sociales. Y, como parte de las soluciones, los cambios en nuestros estilos de vida, el mantenimiento de medidas restrictivas a la interacción, la conciencia de nuestra fragilidad humana y la obediencia sobre las medidas que surgen cada día de acuerdo al significado y momento de la pandemia.

La participación de todos en un proceso global como la pandemia, al menos la sensación de esa participación, ha provocado que, en lo local, en lo cercano y más familiar, de múltiples maneras estemos siendo afectados en nuestras vidas cotidianas. El individuo más humilde y menos visible en la estructura social, tiene su propia interpretación desde su experiencia de participación y es en estos ámbitos de significado personal, desde donde logramos interpretar la intimidad del proceso de aislamiento, confinamiento y distanciamiento, en un momento específico de la trayectoria misma de la pandemia.

La posibilidad de enfermar y la probabilidad de sufrir graves efectos físicos y mentales por el contagio, se ha constituido en una marca significativa en la vida presente de millones de personas modificándose las pautas de conducta, de interacción y de proyección respecto al presente y al futuro de la sociedad, de la familia, del trabajo, de los espacios relacionales y de la manera en que interpretamos nuestro mundo social. El objetivo central fue analizar, desde la perspectiva de las personas, esos cambios individuales y grupales y las propias trayectorias provocadas por las medidas de disminución de la interacción social en la población de Bucaramanga, Colombia, centrando la atención en el primer periodo de aislamiento y confinamiento, desde la declaratoria de emergencia sanitaria en el país desde comienzos del mes de marzo de 2020 y las medidas subsecuentes^10^.

## Materiales y Métodos

La investigación se orientó desde un enfoque cualitativo de carácter fenomenológico, en donde se buscó una comprensión desde las percepciones que las personas le otorgan a la experiencia vivida por la pandemia del COVID-19, especialmente en la fase más intensa de aislamiento y de confinamiento en Colombia^11^. Se pasa entonces por un proceso de descripción, comprensión e interpretación, buscando develar las estructuras de significado desde los sujetos.

Para la realización de la investigación se aplicaron 45 entrevistas en profundidad en total, donde el 60.0% del total corresponden a mujeres (27 entrevistas) y el 40.0% restantes, se aplicaron a hombres (18 entrevistas), la guía de entrevista, estructurada a partir de ejes que incluyeron categorías centrales sobre la experiencia del aislamiento, el confinamiento, la interacción social, entre otras, se aplicó a todos los informantes de manera básica pero manteniendo un margen flexible de producción del discurso según cada contexto de conversación, para la selección de los participantes, se consideraron criterios de inclusión como la mayoría de edad, la distribución por nivel socioeconómico y por diversidad de escolaridad.

La edad promedio de los participantes fue de 44,8 años de edad (20 años el más joven y 70 años el de mayor edad). Adicionalmente, se consideró importante lograr una representatividad por estrato socioeconómico de la vivienda de los informantes, logrando la participación de personas que viven en estrato 1 (17,8%), estratos 2 y 3 (20,0% cada uno), estrato 4 (26,7%) y en los estratos socioeconómicos más altos, es decir 5 y 6, se tuvo una participación del 15.5% restante.

La población también fue diversa en escolaridad, siendo los grupos más representativos, aquellas personas que obtuvieron estudios segundarios y de educación superior. Por estado civil, la gran mayoría, 68,9% son casados o viven en unión libre y, por orientación religiosa, el 77,8% son católicos y el 13,3% afirmaron no tener ninguna orientación de tipo religioso.

Esta breve caracterización de los participantes en este estudio, desde un método de muestreo intencional, se realiza buscando cubrir un amplio abanico de lugares y condiciones sociales para una lectura interpretativa de las experiencias en la pandemia que permita abordar la complejidad misma de las percepciones sociales sobre los cambios en la pandemia.

Las entrevistas se realizaron previa aceptación de cada participante del consentimiento informado avalado por el Comité de Ética de la Universidad, la aplicación de las entrevistas se llevó a cabo a través de medios tecnológicos, privilegiando las entrevistas cara a cara, a través de plataformas como Zoom o Teams, buscando la interacción necesaria entre entrevistador y entrevistado y la aplicación total de la guía de entrevista diseñada por el equipo de investigación. Finalmente, las transcripciones de las entrevistas se analizan con ayuda del software Atlas.ti®, la base de datos fue almacenada en Mendeley Data^12^.

## Resultados

### El encierro: “Todo es costumbre y es por un bien”

Un aspecto importante al considerar el análisis de las situaciones desencadenadas con las medidas de aislamiento y de confinamiento, es la resignación colectiva al mandato de unas medidas sin precedentes en la historia contemporánea. El cálculo de las situaciones conlleva el racionalismo colectivo de las justificaciones y de las consecuencias del encierro, la persistente argumentación médica detrás de las imposiciones políticas, se asimilan desde la promesa la seguridad individual y familiar, mecanismo útil para la aceptación y resignación ^6^. No obstante, los relatos biográficos en esta ruta de vida, dan la sensación de un proceso difícil, pero asimilable por la aceptación y de acomodamiento a la nueva situación, frente a la velocidad de contagio y letalidad del virus.

*El hecho que nos mandaran a encerrar me impactó mucho, los primeros tres días de encierro yo lloré mucho, por una ventana de mi cuarto yo podía ver hacia la calle, no se veía a nadie, tampoco a ningún carro, ver tanta soledad me dio mucha tristeza y me ponía a llorar. (M20)*

*La vida cambia en muchos aspectos: la parte del contacto físico, el aislamiento que en ese momento uno sabe para qué es, pero queda como esa secuela de no querer que él se me acerque tanto. Los dos hemos estado aislados por ese temor que vuelva la enfermedad. (M15)*

Otro elemento emergente en la situación de encierro, es la colectivización del propio discurso médico y psicológico entre las personas que buscan ávidamente noticias, información y recursos “científicos” para entender su propia situación y la del resto de la población confinada. Las personas se convirtieron en expertos en pandemia, en virus, en efectos mentales del encierro, también en tecnología de las comunicaciones y en medidas diversas para contrarrestar los efectos, reales e imaginados, del cierre abrupto de la mayoría de interacciones sociales.

*Pienso que más que dar información acerca de enfermos y muertos, se debe dar más información educativa porque pienso que no hubo la suficiente y la gente no tiene conciencia ni conocimiento sobre la propagación del virus. Por la falta de educación sobre el virus por eso es que la gente le da validez a cosas que no son ciertas y cuando ya lo ve cerca es que se da cuenta que si existe. (M21)*

*También sé de muchas personas que sufren de estrés por el tema del encierro, entonces eso también repercute en mí y he sentido con frecuencia dolores de cabeza, estrés, sentimientos de estar enfermo sin estarlo, dolor de espalda (…). (H2)*

*Mi Dios ha sido muy bueno con los colombianos porque la pandemia no ha sido tan acentuada. (H12)*

La experticia colectiva, causada por el encierro obligado, en diferentes temas no solo se alimenta de manera inédita de la información en alta circulación por diferentes canales y medios de comunicación, sino que es también el catalizador de las diversas fábulas, conspiraciones y proyecciones sobre destinos trágicos para la humanidad^13^.

*Yo pienso que nos engañaron, la organización de salud, no nos ha dicho la verdad, no nos han explicado cómo tratar la enfermedad. (M13)*

*Al comienzo no hubo en los medios de comunicación una buena información sobre qué era lo que se tenía que hacer. Por eso es que en China y en España hubo tanto muerto porque en las noticias lo que decían era que no se debía tomar ibuprofeno ni otros remedios que los médicos siempre mandan, lo que hicieron fue como desacreditar el título de los médicos. El colombiano se toma lo que caiga y se toma lo que sea con tal de no ir al médico. (M12)*

El consumo de información, de datos con y sin contexto, alimenta de manera extraordinaria la gran capacidad colectiva de interpretación de las situaciones cambiantes que causó el encierro de grandes conglomerados de población en diferentes lugares del mundo, las personas se convirtieron en analistas especializados o simplemente en consumidores de interpretaciones coyunturales se acuerdo al manejo y énfasis de las cuestiones manipulables y útiles para algún interés en el trascurso de la pandemia.

*Yo pienso que desde arriba es que viene todo ese descontrol. Por ejemplo, yo nunca había visto que un presidente se presentara en radio o televisión y no siendo epidemiólogo se pusiera a hablar y a dictar normas. Pero lo que dice es lo mismo que uno lee en todas partes. Cuando entrevistan a epidemiólogos o leo artículos sobre la pandemia es lo mismo. Pero el Estado pregona una cosa y lo que se hace es otra. (M25)*

*Yo creo que es diferente para cada cultura y no sólo entre países sino también hablando de culturas dentro del mismo país. Por ejemplo, en Bucaramanga yo podría decir que la gente no entiende lo que está pasando, no tienen la educación suficiente para entender lo que es un virus. Yo he visto en la calle gente que se quita el tapabocas para estornudar, entonces las personas cumplen con las normas sólo por evitar una multa, pero no entienden la importancia de eso. (M5)*

Diversas manifestaciones acerca del significado del encierro, se expresan básicamente en un proceso de autoaprendizaje sobre la importancia atribuida a las drásticas medidas que implicaron el aislamiento en los lugares de vivienda. Convivir con la familia, extrañamente, se convierte en algo nuevo, distinto, negociable y que requiere ajustes continuos, dadas las expresiones de nuevas necesidades en el ámbito doméstico, que, supuestamente, ya eran cotidianas antes de la situación.

*En cuanto a las rutinas ahora tengo más trabajo porque los pelaos están todo el tiempo en la casa y tengo que estar pendiente más de las comidas, antes ellos se iban para la calle uno les daba la plata para que compraran comida. Al que si le ha dado más duro el encierro ha sido a mi esposo porque él si tenía que salir más que yo. (M12)*

*Acá nos tocó a cada uno como se dice “marcar terreno” en la casa. Cada uno cogió su cuarto y enciérrese (risas) Mi hija está estudiando una especialización y se encierra en su cuarto cuando está recibiendo sus clases. Yo igual me encierro en mi cuarto a dar las clases porque no crea, cualquier ruidito no crea, desconcentra. Entonces acá en la casa ya saben que estoy en clase y no vienen con el cuento de interrumpir (H4)*

La familia y la vivienda, se convirtieron en ámbitos distintos por descubrir. Acomodarse a la situación por temor a la enfermedad o por temor al mandato del confinamiento, implicó, en términos generales, una resignificación de la manera en que se ven las personas más cercanas, aquellas con quienes se ha vivido en encierro. La situación familiar, en esencia, se modificó, pero no necesariamente en el sentido negativo por los nefastos efectos que se preveían en la vida en el hogar^13^^,^^15^, auque los efectos suelen ser bastante diferenciados según las propias dotaciones educativas, sicológicas y económicas de las familias^16^^,^^17^.

*Luego, entender las dinámicas familiares: desde qué les gusta desayunar, almorzar y comer hasta los horarios de dormir y levantarse. Como mi hermana trabaja aquí en el municipio donde viven mis papás, ella también decidió venirse para acá y aportar para ellos que irse aparte y pagar un arriendo. La convivencia con mi hermana ha sido un poco compleja, aunque somos contemporáneas somos muy diferentes, pero hemos logrado vivir en armonía a pesar de las dificultades. (M21)*

*Digamos también que las relaciones familiares se vuelven un poco más estrechas porque se comparte más tiempo con esa persona. Con todo esto hay dos opciones: o que las relaciones se corten o sean más distantes o como en mi caso se vuelvan más estrechas. También como uno es solo pues las personas más cercanas pasan a ser la red más cercana de uno. (M5)*

**Tabla 1:** Resumen matriz de categorías por niveles de justificación desde los participantes

	El refugio en la familia	Manejo de la información	Colectivización del discurso	Obediencia al mandato
Obediencia al mandato y cambio de la seguridad individual y familiar	Prácticas de supervivencia y reacomodación	Saturación de información en medios y redes de comunicación	Legitimación del discurso del aislamiento y el confinamiento	
Colectivización del discurso médico y psicológico	La condición del autocuidado como práctica de autorresponsabilidad	La vigilancia colectiva al suceso de la pandemia		La seguridad en la evidencia de las consecuencias de la pandemia
Manejo y manipulación de la información sobre la pandemia	La familia y la vivienda como el lugar seguro		La vigilancia del dato epidemiológico, como miedo y esperanza	Incapacidad de distinguir lo objetivo de lo manipulable
El refugio en la familia y la resignificación de la vida doméstica		La cercanía familiar como seguridad aparente del grupo familiar	La disminución del riesgo en lo cercano, lo familiar	Un retorno a lo “normal” depende de la conducta de cada grupo familiar

Es interesante el alto nivel de aceptación colectiva de las medidas de aislamiento y de confinamiento, básicamente bajo el presupuesto de la responsabilidad individual y familiar, como grupos de autocuidado, así como el intensivo uso de la información circulante, de cualquier tipo, fuente e intención, para una justificación informada sobre los supuestos mismo de las medidas más drásticas. El alto nivel de autocuidado significa que, en las dotaciones individuales, las personas rápidamente asumieron incluso medidas que no hicieron parte de los paquetes restrictivos, ampliando continuamente el ámbito de las restricciones en la vida doméstica.

### El encierro: “(…) ahora lo veo como algo muy normal”.

Las múltiples manifestaciones sobre el significado del aislamiento y del confinamiento, se traducen en la expresión sobre el encierro obligatorio. Cerrar las vidas, implicó cerrar los cuerpos, encerrar los movimientos y limitar la interacción, incluso entre los propios miembros de la familia en espacios muy reducidos, se produce bajo el convencimiento de que es la única manera, la única medida posible en los primeros meses de la pandemia, aunque sin una garantía de protección real.

*Yo creo que la gente resistió lo que más podía, hubo un manejo para mí equivocado por parte del gobierno porque cerró iniciando y abrió cuando el Covid ya estaba regado, entonces ya vamos 4 meses en meseta, de los países con mayor tiempo, o sea nosotros no hemos vivido el pico bajo, y cada vez son más muertos, más familias afectadas, la gente no puede quedarse en la casa, porque por el encierro de tanto tiempo empezó a tener problemas mentales terribles. (H17)*

*Al comienzo uno se sentía muy encerrado, como en una prisión. Pero ahora lo veo como algo muy normal. (H1)*

El trabajo y las actividades económicas que las personas realizan, constituyen el eje más importante sobre el proceso de afectación y de cambios en la vida cotidiana, tanto de las personas como de sus grupos familiares confinados. Las restricciones a la movilidad, el trabajo en casa, el uso intensivo de tecnologías, son solo síntomas de los impactos, positivos y negativos, que conllevó esta medida sin precedentes contemporáneos por su generalización a nivel mundial. Las personas hacen balances positivos sobre la autogestión de tiempos e intensidades de la actividad laboral, pero, también, las personas señalan bruscos cambios en este sentido, las sobrecargas laborales, los trastornos de las rutinas, el uso intensivo de medios tecnológicos, la ansiedad, los cambios las rutinas que permitían una distinción entre tiempo laboral y tiempo de descanso, las drásticas medidas de asepsia, y muchas otras, dan cuenta de una radical transformación en medio del encierro.

*Entonces la organización donde trabajo es humanitaria y por suerte no perdí mi trabajo, pero no me siento bien porque estoy ganando mi salario haciendo nada, es una sensación un poco fea. Pues ha habido algo positivo porque he estado estudiando inglés durante la cuarentena y pues ha sido la forma para aprovechar el tiempo estudiando algo. (H7)*

*Primero, yo salía todos los días, me la pasaba fuera de la casa. Salía a las 6 am y regresaba a las 9 pm por razones laborales. Para mí, fue un cambio bastante brusco. (H1)*

*Ahora me la paso todo el tiempo en el apartamento. Desde marzo cuando la empresa nos mandó a hacer trabajo en casa, lo máximo que he salido a la calle, ha sido a una cuadra. Yo siempre me venía de la oficina a la casa por la tarde a pie. Ahora nada, me la paso todo el tiempo en el apartamento. (M4)*

*Respecto a mi rutina de trabajo tuve que aumentar mi jornada de trabajo Como yo tuve que prescindir de unas personas en el restaurante entonces me tocó a mí hacer de todo. Antes me levantaba tarde y me acostaba temprano entonces ahora es al revés, me levanto temprano y me acuesto tarde porque debo estar más tiempo en el trabajo. (M6)*

En el terreno de lo positivo, además de lo señalado, la ganancia generalizada en los relatos de los participantes, se expresa en la posibilidad de administrar el ámbito de lo doméstico y las relaciones intrafamiliares, especialmente emerge una sensación de diálogo, de compartir espacios y actividades que antes eran relegadas a espacios y tiempos muy marginales.

*Pero creo que también se han generado dinámicas más positivas a nivel familiar porque ha generado más convivencia. (M21)*

*Tenemos tiempo de hablar de dialogar con nuestros hijos, pues muchas veces, uno se iba a trabajar y cada uno por su lado, uno en su trabajo y los hijos estudiando. Uno llegaba a la casa a descansar y ellos con sus trabajos y ni hablaba. Y uno ahora se da cuenta con la pandemia que uno necesita a la familia, entonces, uno habla más. Yo creo que hay más comunicación, hay más como calor de familia, más unión. Jugamos parques, vemos una película, hacemos un juego. (M24)*

*Mi estado emocional ha estado bien porque estoy acompañado de mi familia, practicamos muchas actividades. Por internet colocamos ejercicios y los hacemos todos, juegos, películas de Netflix. (H1)*

*Fue muy eficiente la respuesta de mis familiares, de mis hijos y la esposa de mi hijo mayor estuvieron pendientes a todo momento: mañana, tarde y noche. Entonces eso me dio mucho valor y deseos de recuperarme, me dio mucha moral. (H10)*

*Como vivo sólo con mi esposa, ya nuestras hijas se fueron, teníamos una vida muy normal, el tapabocas lo usábamos sólo para salir de casa y como estábamos siendo rigurosos con las otras medidas entonces dentro de la casa no lo usábamos. Pero resultó una sobrina que empezó haciendo actividades para encuentro familiar, pues es muy habilidosa para el manejo de la tecnología y pues todas esas actividades de cumpleaños y otros encuentros familiares los empezamos a realizar por estas plataformas virtuales y pues nos dimos cuenta que eso funciona, entonces durante todo el año las hemos realizado así, para nosotros no fue mayor problema, pero uno si ve en los más jóvenes que están cansados del encerramiento y que quieren salir. (H18)*

**Tabla 2:** Resumen matriz de categorías por normalización del encierro

	Balance del Encierro	Cerrar y Encerrar
Cerrar los cuerpos, encerrar los movimientos y limitar la interacción	La única opción es el aislamiento, el confinamiento, el resultado depende de cada individuo	
Balance positivo, balance negativo, algo ha cambiado		Mejoramiento en las relaciones interpersonales, especialmente entre el grupo de referencia doméstica. El encierro es cansancio soportable temporalmente.

Los balances individuales sobre el inicio del encierro desde la implementación de las medidas de aislamiento y de confinamiento en los primeros meses de la pandemia, hacen referencia a la manera en que cada individuo y grupo familiar encerrado, asumen y justifican las diferentes medidas externas y, además, como fueron construyendo sus propias fortalezas domésticas luchando contra el contagio.

De una manera generalizada, los relatos nos muestran una enorme capacidad de asimilación de las normas externas y una capacidad mayor en la creación individual de las propias normas de convivencia, seguridad y autocuidado. En la gran mayoría, los balances son bastante positivos en cuanto las relaciones laborales y económicas, también en cuanto a las relaciones con otras personas y con sí mismos. Las enormes afectaciones a la salud, a las finanzas y a las relaciones sociales, se compensan individualmente con esa capacidad de encontrar nuevos significados para viejas interacciones, como la resignificación de la vida doméstica.

## Discusión

El aislamiento y el confinamiento como medidas de choque para mitigar el alto contagio por COVID19 en los primeros meses del año 2020, escaló a nivel mundial, en diferente grado e intensidad, tuvo su primer marco de justificación como medidas necesarias frente a las características epidemiológicas del virus^8^ y la incapacidad aún presente, y durante un largo tiempo, de una respuesta médica efectiva y colectivizada^11^.

La declaración de una pandemia y las subsecuentes medidas, escalaron sin precedentes en todas las sociedades que reaccionaron de manera diferencial6, pero, en todos ellos, antepusieron la responsabilidad individual a través del autocuidado y el encierro en grupos familiares que modificaron las maneras más regularizadas de la interacción social, como lo muestran algunos estudios^11^^,^^13^^,^^18^.

El éxito de estas medidas, no puede aún evaluarse puesto que la pandemia sigue presente en todo el mundo, aunque los anuncios y el inicio de la inoculación masiva con diferentes vacunas apoye el deseo colectivo de un retorno, gradual o total, a lo que se consideraba normal en esa interacción social^18^.

Durante el proceso de aislamiento y confinamiento, especialmente en los primeros meses del año 2020, donde el encierro tuvo su mayor expresión de medidas drásticas, las personas adaptaron sus condiciones y, en general, tuvieron un buen manejo de las circunstancias externas impuestas y de las condiciones domésticas reconstituidas para el relacionamiento con las personas que constituyeron esos grupos de encierro^14^.

Especialmente, llama la atención, desde un balance participativo sobre ese momento de mayor intensidad del encierro, que, a pesar de la sensación de miedo, de alejamiento, de afectación a la salud, a la economía y a vida comunitaria^1^^,^^3^, existe una enorme capacidad de las personas en su vida práctica de resignificar el espacio habitado y las relaciones existentes pero que debieron ser modificadas para enfrentar las situaciones de pandemia, elemento clave para la regularización de la vida social para la superación de la emergencia^4^.

De esta manera, se puede comprender que, pese a los nudos críticos y circunstancias específicas de un alto impacto para las personas y sus familias, la pandemia alecciona sobre la capacidad de reconstitución de la interacción social pese, incluso, a la alta incertidumbre por el futuro inmediato, con un sentido de alta ganancia individual y familiar frente al duro proceso de sentirse parte de un riesgo colectivo tan grande que alcanzó hasta el último resquicio de intimidad de las personas en todo el mundo, aunque una limitante importante en el presente estudio es precisamente la imposibilidad de un estudio etnográfico en los contextos familiares y relacionales en el confinamiento.

## Conclusiones

Las personas, desde diferentes lugares en la estructura social, dan cuenta de un interesante proceso de afrontamiento y de reacomodación de las interacciones sociales, enmarcadas, especialmente, en el ámbito de lo doméstico, pero ampliado a la reconstitución de las relaciones sociales de mayor importancia como el trabajo, las actividades económicas y de relacionamiento social.

El ámbito de lo doméstico constituye, entonces, el lugar de contención y de ordenación para la resignificación de esas pautas de interacción social donde la el trabajo, la familia y las pautas vinculantes con otros, se ven seriamente modificadas, en sentido positivo y negativo, desde la propia perspectiva de las personas que han transitado desde diferentes posiciones sociales por un encierro de alta intensidad en los primeros meses de la pandemia en Colombia.

El drástico encierro de la población colombiana a partir de las medidas transitorias, conlleva una alta incertidumbre de los grupos familiares, pero también una respuesta generalmente positiva y consistente con las medidas de autocuidado que permitieron una cierta sensación de mayor seguridad en el acatamiento de las normas, reales o imaginadas, para enfrentar una pandemia inédita para la población contemporánea.

Las estrategias más comunes tienen que ver con la negociación de las interacciones íntimas, las relaciones domésticas y la asimilación de los cambios que día a día presionan a los grupos familiares respecto a su comportamiento y a su relacionamiento.
